# Cyberbullying victimization identification and large language model-assisted assessment: a study of cyberbullying victimization lexicon construction and validation

**DOI:** 10.3389/fpsyt.2026.1826042

**Published:** 2026-06-24

**Authors:** Xingyun Liu, Yuehan Liao, Fan Feng, Yiming Tu, Xin Kang, Miao Liu, Nuo Han

**Affiliations:** 1 Key Laboratory of Adolescent Cyberpsychology and Behavior(CCNU), Ministry of Education, Wuhan, China; 2 Key Laboratory of Human Development and Mental Health of Hubei Province, School of Psychology, Central China Normal University, Wuhan, China; 3 Huangshan Tunxi No.3 Junior High School, Huangshan, China; 4 Department of Psychology, Faculty of Arts and Sciences, Beijing Normal University at Zhuhai, Zhuhai, China; 5 Beijing Key Laboratory of Applied Experimental Psychology, National Demonstration Center for Experimental Psychology Education (Beijing Normal University), Faculty of Psychology, Beijing Normal University, Beijing, China

**Keywords:** cyberbullying victim, DeepSeek, dictionary, GPT, large language models, social media big data

## Abstract

**Introduction:**

Cyberbullying poses a global mental health threat, yet its accurate identification remains challenging due to biases in self-reporting and help-seeking barriers.

**Methods:**

Based on large-scale social media data, the present study constructed a Chinese cyberbullying victimization lexicon from three dimensions—cyberbullying methods (the types of cyberbullying experienced by the individual), perceived harm (the harm perceived by the victim), and coping strategies (the behavioral responses adopted by the victim)—using Weibo texts, psychological lexicons, and cyberbullying questionnaires. This approach aims to improve the precision of victim identification and facilitate timely intervention. Lexicon validity was evaluated by examining correlations between word-frequency statistics derived from 500 Weibo posts and expert ratings (n = 3). In addition, based on 3,442 RedNote posts, we preliminarily explored the lexicon's cross-platform applicability. To assess whether DeepSeek-R1 and GPT-4o could function as research assistants in dictionary construction, the present study replicated the manual lexicon development process—including text classification, vocabulary screening, weight assignment, and victimization severity assessment—and compared model outputs with human evaluations under simple and complex prompts using Cohen’s Kappa, intraclass correlation coefficients (ICC), recall, and precision.

**Results:**

(1) The lexicon comprised 442 words across three dimensions: cyberbullying methods, perceived harm, and coping strategies. The lexicon demonstrated strong validity in identifying cyberbullying victimization expressions in social media text across each of the three sub-dimensions and the overall dimension (cyberbullying methods: r=0.500, p < 0.001; perceived harm: *r*=0.408, *p* < 0.001; coping strategies: *r*=0.509, p < 0.001; overall cyberbullying victimization expression: *r* = 0.870, *p* < 0.001), and effectively assessed the degree of cyberbullying victimization (*r* = 0.533, p < 0.001); (2) While DeepSeek-R1 performed well on small-scale text classification (Kappa = 0.775–0.781), both models showed significant limitations in large-scale processing (12,600 entries). Vocabulary selection and weight assignment tasks revealed substantial discrepancies with human evaluation (Kappa as low as -0.874), though DeepSeek-R1 achieved moderate consistency in dimension partitioning (Kappa = 0.780).

**Discussion:**

As the first Chinese cyberbullying victimization dictionary, this research demonstrates that large language models offer preliminary utility in structured tasks but require human oversight for complex, large-scale research phases, supporting a human-machine collaborative approach for optimal outcomes.

## 1 Introduction

Cyberbullying constitutes a negative life event and simultaneously serves as a potent source of social stress (1). Extensive research has confirmed its detrimental effects on individual mental health. For instance, Zhao et al. (2) found that experiences of cyberbullying readily trigger negative emotional experiences among adolescents characterized by low threshold, high emotional intensity, and slow recovery to baseline. Furthermore, cyberbullying victimization mediates the relationship between cyberbullying victimization and non-suicidal self-injury. Cyberbullying victimization is also an important predictor of suicide-related behaviors (3). However, cyberbullying victims often refrain from seeking help from authorities due to feelings of shame, fear of retaliation, or belief that seeking help will be ineffective (4). Meanwhile, social psychological service resources are limited (5), resulting in many victims being unable to receive timely professional psychological support after experiencing cyberbullying. Therefore, early proactive identification of cyberbullying victimization and timely provision of psychological intervention are of critical importance.

Despite the rapid development of cyberbullying research, no consensus has yet been reached regarding the definition of cyberbullying (6–8), with different scholars offering varying interpretations and definitions from distinct perspectives. Some researchers simply describe cyberbullying as bullying conducted through technology and online environments. For example, Kowalski and Limber (9) defined cyberbullying as “bullying via email and instant messaging, in chat rooms, on websites, or through text messages sent to mobile phones.” Other researchers have developed definitions by extending traditional bullying concepts. For instance, Hinduja and Patchin (10) assert that “cyberbullying is the intentional, repeated harm inflicted upon another through electronic means.” Correspondingly, cyberbullying victimization is understood as the phenomenon where individuals experience deliberate and repeated electronic harm from others online (11).

However, due to cyberbullying’s complexity and significant variations in victimization rates across studies, Alipan et al. (12) advocate for a multi-perspective, comprehensive definition encompassing perpetrators, victims, and bystanders to establish a multi-layered framework. Alipan et al. define cyberbullying as the direct or indirect use of digital technology targeting one or more individuals. From the victim’s perspective, it is an intentional and harmful act. When the victim perceives both the bully’s malicious intent and the resulting harm, they become a victim of cyberbullying. Given this study’s focus on cyberbullying victims and their expression of victimization, this paper adopts the victim-centered definition proposed by Alipan et al. (12). This definition emphasizes that an individual qualifies as a cyberbullying victim when they perceive the perpetrator’s actions as having clear malicious intent and genuinely experience harm.

The above definition contains two conditions: the victim has been subjected to some form of cyberbullying (such as harassment or cyberstalking), and the victim genuinely feels harmed. Specifically, cyberbullying behaviors may manifest as abusive language (e.g., verbal abuse, insults, and name-calling), offensive language (e.g., obscene expressions, offensive claims, or profanity), threat or blackmail, defamation, and other forms (13, 14), all of which are intended to inflict harm or discomfort on others (15). Following cyberbullying victimization, individuals commonly experience a range of psychological and emotional consequences, including lowered self-esteem, reduced life satisfaction, loneliness, anxiety, and depression, as well as more severe outcomes such as self-harm and suicidal ideation (16–21). In other words, these psychological and emotional consequences reflect the harm perceived by the victim rather than the cyberbullying behavior itself. Meanwhile, victims may adopt coping strategies such as seeking support, avoidance, or retaliation (22). Prior research has shown that coping strategies significantly influence the harm perceived by victims (23), and many studies have examined victims’ coping responses (23–26). Accordingly, the present study identifies cyberbullying victimization expressions on social media from three dimensions: cyberbullying methods, perceived harm, and coping strategies.

Public social websites, such as Twitter, Facebook, Instagram, and Weibo, are the most common platforms for cyberbullying (27). Consequently, these platforms accumulate vast amounts of text, images, audio, video, and other data related to cyberbullying. Furthermore, social media data is publicly accessible, enabling real-time collection and processing of all posts. This facilitates the potential for automated detection of cyberbullying. Most researchers currently use Natural Language Processing (NLP) techniques to annotate and preprocess bullying and hate speech on social media. These techniques are then combined with deep learning, machine learning, or hybrid technologies to train models for the automatic detection of cyberbullying (28–30).

However, cyberbullying not only involves direct verbal attacks from the bully but may also be closely related to the victim’s personal perceptions (31). Therefore, relying solely on the detection of negative statements by the bully fails to comprehensively identify all forms of cyberbullying and is insufficient for timely and effective intervention. Current cyberbullying detection typically classifies roles into bullies and non-bullies (27, 32). However, the non-bully category includes both victims and bystanders, with few studies exploring the identification of cyberbullying victims. Jacobs et al. (33) used NLP techniques to analyze language patterns and content in social media texts to identify bullies, victims, and bystanders. Shoeibi et al. (34) combined keyword and sentiment analysis with machine learning and data mining techniques to automatically detect and identify cyberbullying victims in tweets. Wright and Wachs (35) studied the emotional responses of adolescents facing cyberbullying, identifying potential victims through sentiment vocabulary used on social media.

Although these studies, leveraging NLP technology to identify victims in social media texts, have achieved some success. However, because keyword selection in existing approaches primarily relies on expert knowledge, it lacks a systematic framework and remains limited in scalability and computational performance when identifying cyberbullying victims (36). In contrast, dictionary-based approaches employ theory-driven dimensional classification, corpus-based vocabulary expansion, synonym supplementation, and weight assignment to construct a more comprehensive and structured linguistic feature system (36). Moreover, dictionaries can cover cases not represented in the training data, thereby enhancing the external generalizability of machine learning classifiers (37); building models based on a dictionary can also improve model recognition performance (38). Although NLP technologies based on pre-trained models have become the mainstream in current research, dictionary-based methods typically suffer from limitations such as time lag, weak generalization capabilities, and insufficient contextual semantic understanding (39). Nevertheless, compared to other sentiment analysis techniques, dictionary-based approaches still offer advantages, including high interpretability, low computational cost, and fast processing speed (40). Previous studies have demonstrated the effectiveness of dictionary-based approaches in social media text analysis (38, 41, 42). Yet there is currently no specific dictionary for cyberbullying victimization.

This study defines expressions of cyberbullying victimization as language produced voluntarily by victims on social media platforms after experiencing cyberbullying. These expressions describe their experiences (methods of cyberbullying), subjective feelings (perceived harm), and coping strategies. By compiling a cyberbullying victimization lexicon based on these three dimensions, the study aims to identify victims more comprehensively and promptly, thereby providing them with psychological support and intervention.

However, dictionary compilation requires significant human resource investment, but existing studies have demonstrated that Large Language Models (LLMs) can analyze and distinguish text content from social media to assist researchers with part of their work. For example, LLMs have been used to support preliminary clinical diagnosis, literature screening, and text classification tasks, reducing manual workload while maintaining relatively high accuracy or recall (43–45). In addition, ChatGPT has shown potential in identifying public attitudes based on social media text (46). However, no research has yet explored whether LLMs can perform equally well in dictionary compilation.

DeepSeek-R1 and ChatGPT-4o are currently among the most widely used large language models. At the time of evaluation, ChatGPT represented OpenAI’s most advanced text-based model, featuring stronger contextual understanding and lower operating costs (47). Among the top 40 generative artificial intelligence (AI) tools ranked by traffic, ChatGPT accounts for 82.5% of the total traffic (48), substantially surpassing other models in terms of market share. This dominance may be partially attributable to its performance advantages. Prior research has indicated that ChatGPT demonstrates superior response accuracy and completeness compared to other models such as Gemini and Claude (49). Furthermore, existing studies have shown that ChatGPT-4o exhibits strong overall performance in complex tasks—including accuracy, reliability, and reproducibility—outperforming both Gemini and Claude (47, 50). DeepSeek-R1, developed by a Chinese AI company, further improves inference efficiency while reducing cost compared with previous versions (51). In text classification tasks, DeepSeek generally outperforms Gemini, GPT, and Llama, and although it slightly underperforms Claude, its cost is substantially lower; moreover, Claude’s usage cost is considerably higher than both DeepSeek and GPT (52). In addition, DeepSeek performs better in Chinese-language contexts (53). For example, compared with GPT, DeepSeek has demonstrated greater stability and applicability in Chinese text adaptation tasks (54). Based on these considerations, the present study further explores the potential of ChatGPT and DeepSeek to assist or partially replace manual labor in dictionary compilation. These two models were selected because they represent advanced and widely accessible LLMs with favorable performance-cost tradeoffs and reflect distinct domestic and international technological development backgrounds.

Accordingly, this study aims not only to develop a Chinese lexicon for identifying cyberbullying victims, but also to address the more forward-looking research question of whether large language models can participate in the construction of complex psychometric tools. This study makes two primary contributions. First, it systematically constructs the first Chinese cyberbullying victimization lexicon from the victim’s perspective, providing an interpretable, low-cost, and scalable tool for automated identification of cyberbullying victimization in social media contexts. Second, it systematically evaluates the capabilities of GPT-4o and DeepSeek-R1 across the full workflow of psychological lexicon construction, offering the first empirical examination of the applicability boundaries of LLMs in human-AI collaborative dictionary construction and providing practical evidence for future AI-assisted psychometric tool development. This study has been approved by the Institutional Review Board (IRB) of Central China Normal University (CCNU), with IRB approval number CCNU-IRB-202311046b, confirming that the study complies with ethical standards for human subjects research.

Based on the above research objectives, this study comprises two sub-studies: Study 1 aims to construct and validate a Chinese cyberbullying victimization lexicon to test its effectiveness in identifying victimization expressions and assessing victimization severity; Study 2 further evaluates the feasibility and applicability boundaries of mainstream LLMs in assisting dictionary construction. Accordingly, the following hypotheses are proposed: H1: The cyberbullying victimization lexicon can identify cyberbullying victims and assess the degree of victimization, with its results showing a significant positive correlation with expert ratings; H2: DeepSeek-R1 will outperform ChatGPT-4o in assisting the construction of a Chinese cyberbullying victimization lexicon.

## 2 Study 1: construction and validation of a cyberbullying victimization dictionary

In summary, Study 1 aims to construct a cyberbullying victimization dictionary based on social media data and validate its effectiveness in identifying cyberbullying victim expressions and the severity of victimization, providing an innovative method for victim identification and improving the accuracy of victim detection.

### 2.1 Construction of a cyberbullying victimization dictionary

#### 2.1.1 Methods

##### 2.1.1.1 Data collection

The data used in this study were sourced from Sina Weibo. As of March 2025, Sina Weibo had 591 million monthly active users, with 261 million daily active users. The collected data primarily includes users’ online behaviors (such as reposts and replies) and Weibo post content. Throughout the data collection process, the researchers adhered to privacy and ethical principles, ensuring that all data were anonymized and privacy was strictly protected (55).

Participant Selection Process:(1)Initial identification of users was conducted through public media reports related to cyberbullying victims on Weibo or keyword searches (e.g., “online violence,” “cyberbullying,” “verbal abuse,” “defamation,” “rumors,” “insults,” etc.), resulting in the identification of 82 Weibo users who may have experienced cyberbullying; (2) After downloading and anonymizing the Weibo posts of the 82 initially identified users, four researchers independently reviewed all posts of each user to determine whether they could be classified as victims and to identify the time of their first experience of cyberbullying. Prior to the evaluation, all four researchers received detailed training on the definition, types, and manifestations of cyberbullying. Based on the training content and the inclusion/exclusion criteria listed below, the researchers independently assessed whether each user could be classified as a victim. The inclusion criteria: (1) the user has experienced cyberbullying; and (2) the user has expressed the harm they felt in their posts. The exclusion criteria: (1) the time of the first cyberbullying event could not be determined; (2)Weibo data before and after the first cyberbullying event could not be retrieved (e.g., due to insufficient number of posts or visibility restrictions such as ‘display only posts from the past six months’); (3) highly publicized Weibo users, such as celebrities. Users were only included in the victim group if at least three researchers agreed on their classification as a cyberbullying victim. A total of 35 users were included in the victim group, consisting of 11 males and 24 females, and their Weibo posts since registration were downloaded. Given that a small number of victims may be constrained in expressing experiences of cyberbullying victimization, this study further randomly selected 1,000 regular users (227 males, 773 females) to examine their expressions (36). These 1,000 users were drawn from a customized Weibo database comprising 1.06 million active Weibo users (56). Following the methodology of Lv et al. (36), researchers downloaded the two most recent Weibo posts for each expanded user. Ultimately, this study obtained a total of 12,600 Weibo posts, comprising 10,600 posts from 35 cyberbullying victims and 2,000 posts from 1,000 expanded users.

##### 2.1.1.2 Steps

###### 2.1.1.2.1 Step 1: selection of initial words

In the process of constructing the cyberbullying victimization dictionary, 50 posts were randomly selected from the 12,600 Weibo posts, and independently coded by four researchers to determine whether each post was related to cyberbullying victimization. The results showed that the coding had good inter-rater reliability (Kappa = 0.85). The 12,600 posts were then divided into four groups, with each researcher selecting posts related to cyberbullying victimization and relevant vocabulary. After researcher screening, 132 of these Weibo posts were identified as involving cyberbullying victimization, accounting for approximately 1.05% of the total posts. Following standard dictionary compilation methods, each researcher also extracted relevant words from five commonly used cyberbullying measurement scales, including the Cyberbullying Victimization Scale (CVS) (57, 58), Cyberbullying Inventory (CBI) (59, 60), Revised Cyberbullying Inventory (RCBI) (61), Cyberbullying Victimization Questionnaire (CVQ) (62, 63), and the self-developed Cyberbullying Victimization Questionnaire (64), which served as important sources for the initial vocabulary, enhancing the dictionary’s coverage and recognition capability in real-world contexts. The specific details of these scales are as follows:

Cyberbullying Inventory (CBI), developed by Erdur (59) and adapted into Chinese by Zhou et al. (60), consists of two subscales: cyberbullying and cybervictimization, each containing 18 items used to measure the extent of bullying or being bullied online over the past six months. The scale uses a 4-point Likert scale (1 = “never encountered,” 4 = “5 times or more”). Higher scores indicate greater involvement in cyberbullying behaviors. The scale has good reliability and validity in Chinese populations (60).

Revised Cyberbullying Inventory (RCBI), adapted from CBI by Topcu and Erdur-Baker (65) and revised by Chu and Fan (61), contains two subscales: cyberbullying and victimization. It includes 14 items per subscale, using a 4-point scale (1 = “never encountered,” 4 = “more than 3 times”). Higher scores indicate deeper involvement in cyberbullying. The scale has good reliability and validity among Chinese university students (66).

Cyberbullying Victimization Scale (CVS), developed by Çetin et al. (57) and adapted by You (58), includes three dimensions: cyber verbal bullying, concealed identity, and online fraud, with a total of 17 items. It measures individuals’ cyberbullying victimization over the past year using a 5-point Likert scale (1 = “never occurred,” 5 = “always occurred”). Higher scores indicate higher levels of victimization. This scale has demonstrated good reliability and validity in Chinese university students (58).

Cyberbullying Victimization Questionnaire (CVQ), developed by Kwan and Skoric (63) and adapted into Chinese by Chen et al. (62), includes 17 items measuring the extent of cyberbullying victimization over the past year. It uses a 6-point scale from 1 (never occurred) to 6 (more than 10 times), with higher scores indicating more severe victimization. This scale has been widely used in Chinese studies and has demonstrated good internal consistency (67).

The Li Yajun Self-Developed Online Bullying Questionnaire, created by Li (68), includes 9 items measuring different forms of cyberbullying victimization. It employs a 5-point scoring scale (1 = Never, 2 = Only 1–2 times, 3 = 2–3 times per month, 4 = About once per week, 5 = Several times per week). Higher scores indicate greater frequency of online bullying experiences. Previous studies have shown that this questionnaire has good internal consistency in Chinese populations (69).

Liu et al. (20) found that after experiencing cyberbullying, individuals show significant changes in factors such as suicide risk and moral perception. Therefore, researchers also selected words related to cyberbullying victimization from Chinese Suicide Dictionary(CSD) (36), Moral Foundations Dictionary(MFD) (70), and Moral Motivations Dictionary(MMD) (71).

###### 2.1.1.2.2 Step 2: filtering irrelevant and expanding similar words

Additionally, three researchers specializing in cyberbullying research were recruited to independently assess all initial words and determine whether each word was related to expressions of cyberbullying victimization. Words deemed relevant were categorized as “valid,” while others were considered “invalid.” Only words that were rated as “valid” by at least two researchers were retained in the list. During this process, researchers also assigned a weight to each valid word on a scale from 1 to 3, with higher weights indicating greater relevance to cyberbullying victimization. Given that this study focuses on victims of cyberbullying, previous victim studies have also primarily examined the role of victims in the crime, emphasizing victim characteristics, their relationship and interactions with perpetrators, and victim behaviors (72, 73). Consequently, this study categorizes vocabulary into three dimensions: perceived harm (corresponding to victim characteristics), cyberbullying methods (corresponding to victim-perpetrator interactions), and coping strategies after cyberbullying (victim behaviors). Three researchers must determine which dimension each qualified word belongs to; only words agreed upon by at least two researchers remain in the list.

Synonyms were added to the list wherever possible, with these additional words also being evaluated, weighted, and categorized by the three researchers. Words that were agreed upon by at least two researchers were retained in the list. A total of 521 words were retained.

###### 2.1.1.2.3 Step 3: removal of low-frequency and duplicate words

Researchers constructed a Weibo database covering China’s 31 provinces/municipalities/autonomous regions using posts from active users between January 2010 and December 2023. This database was compiled via public API downloads, with necessary filtering criteria applied during data extraction to ensure content validity. For instance, researchers first selected active users registered for over one year with at least 500 total posts. The sample was then narrowed to users with fewer than 3,000 followers, excluding celebrities, professional bloggers, or organizations to focus on ordinary users. Additionally, retweeted posts were excluded from analysis, retaining only original content since retweets do not represent the user’s own expression. Researchers randomly sampled one ten-thousandth of this Weibo database to calculate the frequency of words in the cyberbullying victim dictionary. Terms like “defamation,” “perpetrator,” “indecent photos,” “talking about me,” and “without consent” showed zero frequency and were thus removed. Simultaneously, researchers manually eliminated duplicate words, ultimately removing 79 words in total.

**Figure 1:**
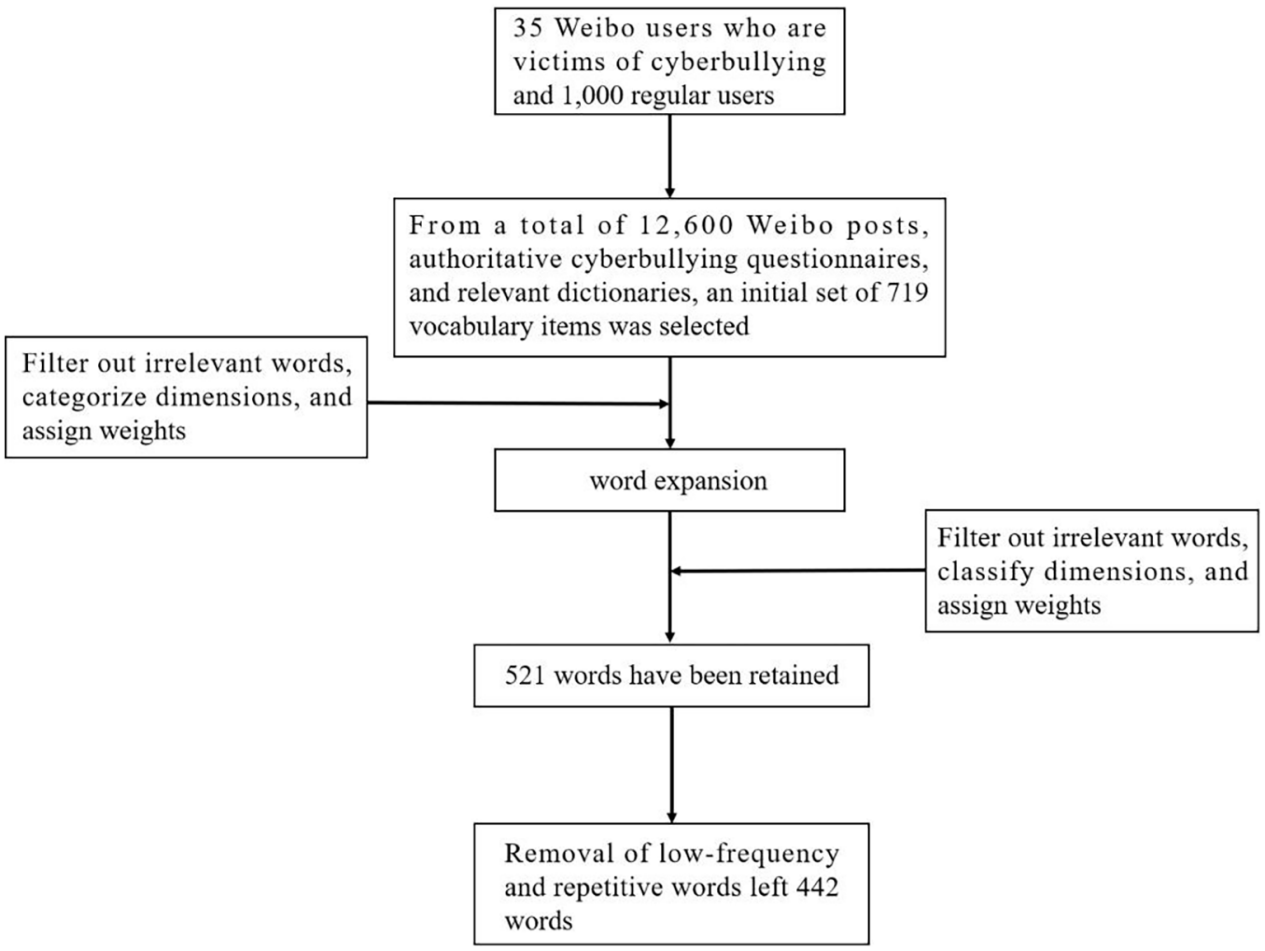
Flowchart of the construction of the cyberbullying victimization.

The final cyberbullying victimization lexicon comprised 442 words across three dimensions—cyberbullying methods (225 words), perceived harm (108 words), and coping strategies (109 words)—which iscomparable to the size of each dimension in dictionaries from previous studies that demonstrated good detection and identification performance (74, 75). The entire process is illustrated in **Figure 1**.

### 2.2 Validation of a cyberbullying victimization dictionary

#### 2.2.1 Methods

##### 2.2.1.1 Data collection

Participant Selection Process: This study distributed an online questionnaire via Weibo, recruiting 497 valid participants, including 129 males and 368 females, with an average age of 24.82 years (SD = 5.34). Exclusion criteria: (1) age under 18 years; (2) fewer than 100 Weibo posts; (3) provision of invalid responses in the online questionnaire; (4) possession of multiple user accounts (different accounts sharing the same IP address).

Cybervictimization Questionnaire (CVQ):The study employed the Cyberbullying Victimization section of the CVQ, originally developed by Kwan and Skoric (63) and revised by Chen et al. (62). This section assessed cyberbullying victimization experiences among the 497 valid participants over the preceding three months. The 17-item scale utilized a 6-point Likert scale, ranging from 1 (never) to 6 (more than 10 times), with increasing frequency levels. Higher total scores indicate more severe cyberbullying victimization. The scale has been widely used in Chinese studies and has demonstrated good internal consistency (67). In this study, the Cronbach’s α coefficient for this scale was 0.93.

Based on the distribution of cyberbullying victimization scores (49.82 ± 16.69), participants were divided into a high-victimization group (CVQ > 66.51) and a non-high-victimization group (CVQ < 33.11). This study randomly selected 30 users from each group, totaling 60 participants (15 males, 45 females) with a mean age of 22.85 years (SD = 3.13). Subsequently, all Weibo posts from these users were collected to construct the “data pools” for the high- and low-victimization groups. Following previous studies (36, 76), 250 posts were randomly sampled from each data pool, resulting in a total of 500 Weibo posts.

##### 2.2.1.2 Steps

Expert ratings were employed as the standard for evaluating the cyberbullying victimization lexicon’s ability to identify expressions of cyberbullying victimization and the severity of such victimization on social media. Consistency between word frequency analysis and expert ratings was used to examine whether the lexicon’s word frequencies could identify Weibo texts expressing experiences of cyberbullying and whether they reflected the severity of victimization among these Weibo users.

Additionally, three researchers specializing in cyberbullying were recruited to evaluate the 500 posts in terms of cyberbullying methods, users’ perceived harm, and users’ coping strategies (these three dimensions jointly constitute cyberbullying victimization expressions), as well as the overall level of cyberbullying victimization. The 500 posts were randomly selected from the 30 users in the high-victimization group and the 30 users in the low-victimization group described in Section 2.2.1.1, with 250 posts drawn from each group’s data pool. All evaluations were conducted using a 5-point Likert scale, where 1 indicates “This Weibo post is unrelated to this dimension”/”the user’s cyberbullying victimization level is very low”. In contrast, 5 indicates “the content of this microblog is highly relevant to this dimension”/”the user’s cyberbullying victimization level expressed in this microblog is very high.” Higher scores indicate more expressions of victimization in that dimension within the microblog or a higher level of cyberbullying victimization for the user. Ultimately, three sets of scoring data were obtained for 500 microblogs.

##### 2.2.1.3 Analysis

This study first aggregated the scores from three researchers across each dimension for each Weibo post to form an overall score. It then calculated the average score from the three researchers for each post across four aspects: cyberbullying methods, perceived harm, coping strategies, and overall score. The average of each post’s overall score served as its total score for cyberbullying victimization expression. Simultaneously, the average score from the three researchers regarding the severity of cyberbullying victimization was calculated as the severity score. Consequently, each microblog corresponds to multiple scoring metrics: scores across dimensions, cyberbullying victimization expression scores, and severity scores. Additionally, researchers utilized the cyberbullying victimization lexicon to calculate the sub-dimensional word frequency and total word frequency for each microblog post, analyzed from three dimensions—cyberbullying methods, perceived harm, and coping strategies—as well as from the perspective of the overall lexicon. Finally, researchers assessed the lexicon’s effectiveness in identifying expressions of cyberbullying victimization by calculating the Pearson correlation between expert ratings and both sub-dimensional word frequency and total word frequency.

#### 2.2.2 Results

The results of the Pearson correlation analysis are presented in **Table 1**. In the 500 Weibo texts, word frequencies in each dimension and the overall word frequency were significantly positively correlated with the expert ratings. These correlation coefficients fell within the range of moderate to strong correlation according to general standards (77). Therefore, these results indicate the reliability of the dictionary in identifying expressions of cyberbullying victimization and assessing the degree of cyberbullying victimization, H1 was supported by the findings of this study.

**Table 1:** Descriptive statistics and correlations between dictionary-based identification and expert ratings.

Dimension	Scoring method	Weibo posts (n)	M	SD	r	p
Cyberbullying behaviors	Expert ratings	500	2.43	1.65	0.500***	< 0.001
	Word frequency	500	2.44	5.89		
Perceived harm	Expert ratings	500	2.24	1.41	0.408***	< 0.001
	Word frequency	500	0.57	1.32		
Coping strategies	Expert ratings	500	2.33	1.52	0.509***	< 0.001
	Word frequency	500	1.30	2.78		
Cyberbullying victimization expression	Expert ratings	500	1.77	1.84	0.870***	< 0.001
	Word frequency	500	4.31	8.86		
Degree of cyberbullying victimization	Expert ratings	500	2.44	1.62	0.533***	< 0.001
	Word frequency	500	4.31	8.96		

*p < 0.05, **p < 0.01, ***p < 0.001.

### 2.3 Exploration of the lexicon’s applicability on other platforms

#### 2.3.1 Methods

##### 2.3.1.1 Data collection

In addition to Weibo, Douyin and RedNote are also major Chinese social media platforms with large user bases. Considering that Douyin is primarily a short-video platform (78), whereas RedNote mainly features image-and-text posts, the present study collected homepage posts from RedNote users to preliminarily explore the applicability of the lexicon on other Chinese social media platforms.

The participant recruitment and screening procedures were as follows: (1) First, potential RedNote users who may have experienced cyberbullying were preliminarily identified through keyword searches within the platform (e.g., “cyberbullying” “online violence” “being insulted” “rumor spreading” “cyberbullying experience” and “being attacked”), public media reports, and posts explicitly describing cyberbullying experiences published by the users themselves. A total of 10 candidate users were initially identified. (2) Subsequently, three researchers independently reviewed all homepage posts of each user to determine whether the user could be classified as a cyberbullying victim. Prior to the formal evaluation, all researchers received standardized training on the definition, types, and manifestations of cyberbullying. Judgments were made according to the following inclusion and exclusion criteria.

The inclusion criteria for the victim group were as follows: (1) the user had experienced cyberbullying behaviors (e.g., The user clearly described in the post that they had suffered from various forms of online attacks, insults, humiliation, rumor spreading, harassment, or other forms of cyberbullying from others); (2) the user expressed perceived harm caused by cyberbullying in posts; and (3) the account was a publicly visible ordinary personal account. The exclusion criteria for the victim group were as follows: (1) the cumulative number of posts was fewer than 100; (2) the account was commercially or institutionally operated; and (3) the user possessed relatively high public visibility or social influence, such as celebrities or internet influencers. A user was included in the victim group only when at least two researchers reached the same judgment. Ultimately, eight victim users were included.

Control group users were also independently screened by three researchers based on the inclusion and exclusion criteria. The inclusion criteria for the control group were as follows: (1) the user had not experienced cyberbullying behaviors; (2) the account characteristics (e.g., number of posts and followers) were generally matched with those of the victim group users; and (3) the account was a publicly visible ordinary personal account. The exclusion criteria for the control group were as follows: (1) the user had experienced cyberbullying behaviors; (2) the user had expressed perceived harm caused by cyberbullying in posts; (3) the account was commercially or institutionally operated; and (4) the user possessed relatively high public visibility or social influence, such as celebrities or internet influencers. A user was included in the control group only when at least two researchers reached the same judgment.

Finally, a total of 16 users were included, including 4 males and 12 females. Subsequently, all homepage posts from both groups were collected, resulting in a total of 3,442 post texts, including 1,846 posts from the victim group and 1,596 posts from the control group.

##### 2.3.1.2 Analysis

The cyberbullying victim lexicon developed in the present study was applied to calculate lexicon word frequencies in RedNote users’ posts across the dimensions of cyberbullying methods, perceived harm, coping strategies, and overall victimization. Specifically, the proportion of lexicon word frequencies in each dimension was calculated for each user, and statistical analyses were conducted at the user level.

Given the relatively small sample size of the present study and the fact that this part of the analysis aims to exploratorily validate the dictionary’s applicability across different Chinese social media platforms, effect size indices were emphasized in addition to significance testing. Specifically, independent-samples t-tests were conducted to compare differences in lexicon word frequencies between the victim group and the control group across all dimensions. Furthermore, Cohen’s d and Hedges’ g were calculated as effect size indicators to evaluate the discriminative ability of the lexicon in identifying cyberbullying victims across different platforms.

#### 2.3.2 Results

The comparison results of lexicon word frequencies between the victim group and the control group on RedNote are presented in **Table 2**. The results showed that the victim group exhibited higher word frequencies than the control group across the three subdimensions of cyberbullying methods, perceived harm, coping strategies, as well as the overall cyberbullying victimization dimension. Among them, significant group differences were observed in the dimensions of cyberbullying methods, perceived harm, and coping strategies, while the overall cyberbullying victimization dimension showed the most pronounced difference.

**Table 2:** Descriptive statistics and group comparisons of lexicon word frequencies across dimensions between the victim group and the control group on RedNote.

Dimension	Victim group		Control group		t	Cohen’s d	Hedges’ g
	M(10-^2^)	SD(10-^2^)	M(10-^2^)	SD(10-^2^)			
Cyberbullying methods	0.45	0.15	0.24	0.12	3.159^**^	1.580	1.493
Perceived harm	0.32	0.18	0.13	0.07	2.738^*^	1.369	1.294
Coping strategies	0.44	0.23	0.22	0.09	2.400^*^	1.200	1.135
Overall cyberbullying victimization	1.20	0.30	0.59	0.14	5.216^***^	2.608	2.465

*p < 0.05, **p < 0.01, ***p < 0.001.

Furthermore, all dimensions demonstrated large effect sizes (Cohen’s d = 1.200 ~ 2.608, Hedges’ g = 1.135 ~ 2.465), indicating that the cyberbullying victim lexicon could effectively distinguish cyberbullying victims from non-victims on the RedNote platform as well. These findings preliminarily support the potential cross-platform applicability of the lexicon across different Chinese social media platforms.

### 2.4 Discussion

**Figure 2:**
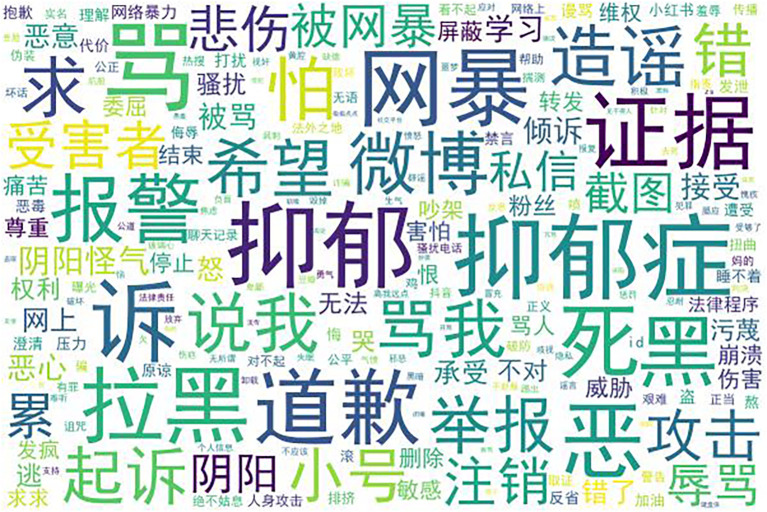
Lexicon word cloud. 抑郁, depression, 网暴, cyberbullying, 抑郁症, clinical depression, 骂, insult, 证据, evidence, 拉黑, block, 诉, complain, 道歉, apologize, 死, die, 恶, malice, 报警, call the police, 举报, report, 攻击, attack, 注销, deactivate, 造谣, rumor, 错, wrong, 怕, fear, 求, beg, 起诉, sue, 小号, burner account, 辱骂, abuse.

This study follows the methods of previous research in constructing dictionaries (36, 76) and systematically constructs a Chinese cyberbullying victim lexicon for the first time. Its validity has been verified to identify cyberbullying victim expressions of Sina Weibo users and measure their victimization level. Meanwhile, based on textual data from RedNote users, the present study further conducted an exploratory analysis of the potential cross-platform applicability of the cyberbullying victim lexicon across different Chinese social media platforms. The results indicate a significant moderate to high correlation between expert ratings and word frequency analysis (*r* = 0.408 ~0.870, *p* < 0.001). This lexicon effectively identifies cyberbullying victim expressions in Weibo texts and measures the degree of an individual’s cyberbullying victimization. Furthermore, the analyses on the RedNote platform demonstrated that the victim group exhibited significantly higher word frequencies than the control group across the three subdimensions of cyberbullying methods, perceived harm, coping strategies, as well as the overall cyberbullying victimization dimension, with all dimensions showing large effect sizes. These findings suggest that although different Chinese social media platforms vary in language style, expression habits, and interaction mechanisms, the lexicon still demonstrates a certain degree of discriminative ability on non-Weibo platforms, thereby preliminarily supporting its potential cross-platform applicability across different Chinese social media platforms. Specifically, the lexicon includes a total of 442 words, divided into three dimensions: Cyberbullying Methods (225 words), Perception of Harm (108 words), and Coping Strategies (109 words). **Figure 2** shows the word cloud generated from 500 microblogs used in the dictionary validation phase. The results reveal that terms such as “depression,” “depressive disorder,” “cyberbullying,” and “insults” appear with high frequency in microblogs subjected to cyberbullying.

Compared to existing research that predominantly focuses on identifying perpetrators’ verbal expressions (28–30, 79), this study shifts its focus to recognizing expressions from the victim’s perspective, thereby expanding the scope of cyberbullying identification research. Furthermore, previous approaches to identifying cyberbullying victims often relied on generic psychological dictionaries, such as LIWC and emotional vocabulary, which demonstrated limited effectiveness and lack of specificity in concrete cyberbullying victimization contexts (33). This study addresses the limitations of existing psychological language dictionaries in cyberbullying contexts by constructing a thematic, highly relevant cyberbullying victimization dictionary through a combined corpus-driven and expert-evaluated approach. This dictionary offers enhanced interpretability and predictive power. Leveraging natural language processing methods, it enables real-time analysis of social media texts, serving as an auxiliary tool in mental health screening or crisis intervention systems to improve screening efficiency and advance the intelligent development of psychological services.

## 3 Study 2: feasibility of GPT and DeepSeek in lexicon construction

Study 2 aims to replicate the lexicon construction process performed by researchers using GPT-4o (released in January 2025) and DeepSeek-R1 (DeepSeek-R1-671B), verifying the feasibility of using large language models (LLMs) to assist researchers in lexicon compilation, thereby reducing the manual effort involved.

### 3.1 Methods

#### 3.1.1 Data collection

The data used in this study are from Study 1.

#### 3.1.2 Steps

Step 1: Identifying Weibo Texts Related to Cyberbullying Victimization. First, GPT-4o and DeepSeek-R1 independently coded 50 randomly selected Weibo posts from Study 1, which were part of a total of 12,600 posts, to determine whether each post was related to cyberbullying victimization. Both models then independently coded the four groups of Weibo posts from Study 1, selecting those that expressed cyberbullying victimization (with “related” coded as 1 and “unrelated” coded as 0).

Step 2: Selecting Initial Words. Words related to cyberbullying victimization were selected from the identified Weibo posts in Study 1(Construction of a Cyberbullying Victimization Dictionary), five scales [CVS (57, 58), CBI (59, 60), RCBI (61), CVQ (62, 63), and Li Yajun’s self-developed Cybervictimization Questionnaire (64)), as well as three lexicons (CSD (36), MFD (70), and MMD (71)].

Step 3: Filtering Unrelated and Expanding Synonymous Words. GPT-4o and DeepSeek-R1 were used to evaluate the initial words extracted from Study 1 (Construction of a Cyberbullying Victimization Dictionary) and additional synonymous words to determine if each word was related to cyberbullying victimization. Words deemed related were classified as “qualified”, while those not deemed related were classified as “unqualified”. Simultaneously, weights were assigned to the “qualified” words, and dimensions were evaluated.

**Figure 3:**
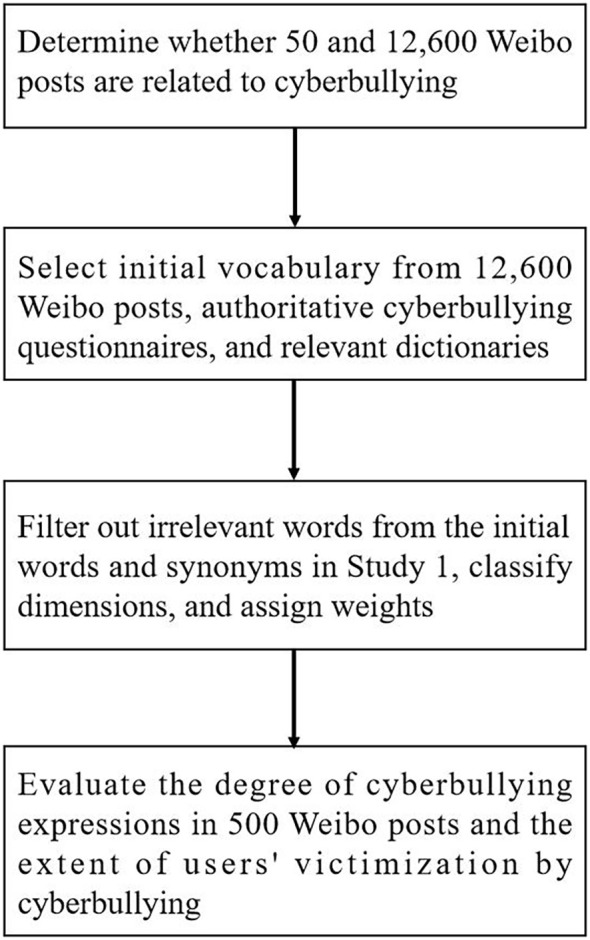
Flowchart of GPT and DeepSeek participation in constructing the cyberbullying victim lexicon.

Step 4: Evaluating Cyberbullying Expression and the Degree of Victimization. GPT-4o and DeepSeek-R1 were employed to analyze 500 Weibo posts from the Validation of a Cyberbullying Victimization Dictionary in Study 1, assessing the extent of cyberbullying methods, harm perception, coping strategies, and the degree of cyberbullying victimization expressed in the texts. The evaluation criteria were consistent with those used in Study 1. A detailed process is shown in **Figure 3**.

All steps above were completed by researchers inputting instructions to GPT and DeepSeek. This study designed two types of prompts based on Silhadi et al. (80): Simple prompts (containing only definitions/explanations and task instructions) and complex prompts (containing role-assignment (e.g., “You are a researcher compiling a dictionary of cyberbullying victimization terms”), definitions/explanations and task instructions). Thus, each step yielded two sets of evaluation results for both GPT-4o and DeepSeek-R1. The prompt content (definitions/explanations and task instructions) input at each step matched the training material provided to researchers in Study 1, as detailed in **Supplementary Appendix 1**.

#### 3.1.3 Analysis

We first calculated the Kappa coefficients and intraclass correlation coefficients (ICC) of the evaluation results from GPT-4o and DeepSeek-R1 compared to the manual evaluations for each step, to assess consistency. Meanwhile, using the manual evaluation results as the ground truth, we calculated the recall rates and precision for the evaluation results of GPT-4o and DeepSeek-R1 in each step to achieve a more precise assessment of whether these models could assist researchers in lexicon compilation, thereby alleviating manual workload.

### 3.2 Results

#### 3.2.1 Classification performance

Subset of 50 Weibo Posts. The DeepSeek model showed high consistency with the researchers’ evaluations, whereas the GPT model demonstrated moderate consistency. The simple prompt outperformed the complex prompt overall (see **Table 3**).

**Table 3:** Consistency between models and researchers in classification.

Dataset/group	Model	Prompt	Kappa	p
Subset of 50 Weibo Posts	DeepSeek	Simple	0.781	<0.001
	DeepSeek	Complex	0.775	<0.001
	GPT-4o	Simple	0.650	<0.001
	GPT-4o	Complex	0.575	<0.001
Dataset of 12,600 Weibo Posts				
Group 1	DeepSeek	Simple	0.048	<0.001
	DeepSeek	Complex	0.040	<0.001
	GPT-4o	Simple	0.136	<0.001
	GPT-4o	Complex	0.153	<0.001
Group 2	DeepSeek	Simple	0.104	<0.001
	DeepSeek	Complex	0.156	<0.001
	GPT-4o	Simple	0.416	<0.001
	GPT-4o	Complex	0.421	<0.001
Group 3	DeepSeek	Simple	0.249	<0.001
	DeepSeek	Complex	0.263	<0.001
	GPT-4o	Simple	\	\
	GPT-4o	Complex	0.089	<0.001
Group 4	DeepSeek	Simple	0.293	<0.001
	DeepSeek	Complex	0.225	<0.001
	GPT-4o	Simple	-0.012	0.512
	GPT-4o	Complex	0.237	<0.001

Dataset of 12,600 Weibo Posts. The consistency between the models and the researchers significantly decreased. DeepSeek performed poorly in the judgment of the 1st to 2nd groups of Weibo posts, and showed better consistency with researchers in the 3rd and 4th groups. GPT (simple prompt) showed very low consistency in the 1st and 4th group evaluations, moderate consistency in the 2nd group, and because GPT (simple prompt) judged all posts in the 3rd group as non-cyberbullying, the Kappa coefficient could not be calculated. Using the researchers’ classification results as the ground truth, GPT (simple prompt) had a recall and precision of 0 for the 3rd group; GPT (complex prompt) showed low consistency with researchers in the 1st, 3rd, and 4th groups, and moderate consistency in the 2nd group (see **Table 3**). In the classification tasks for the 1st and 2nd groups, ChatGPT-4o showed better consistency with researchers than DeepSeek-R1; thus, H2 was not supported.

#### 3.2.2 Dictionary construction

Word Selection. DeepSeek and GPTshowed poor consistency with the researchers in selecting words related to cyberbullying from the 1st to 4th group of Weibo posts, scales, and lexicons (see **Table 4**). Since all words extracted by DeepSeek (complex prompt) and GPT (complex prompt) from the Group 1 had been extracted by the researchers (i.e., all coded as 1), the Kappa coefficient could not be calculated. Using the researchers’ selection results as the ground truth, DeepSeek (complex prompt) had a recall of 15.95% and precision of 100% for words extracted from Group 1, while GPT (complex prompt) had a recall of 5.09% and precision of 100%.

**Table 4:** Consistency between each model and researchers in the four steps of dictionary construction.

Task	Model	Prompt	Kappa/ICC	p
Word selection	DeepSeek	Simple	Kappa = -0.833 ~ -0.217	<0.001
	DeepSeek	Complex	Kappa = -0.768 ~ -0.189	<0.01
	GPT-4o	Simple	Kappa = -0.144 ~ -0.014	<0.001
	GPT-4o	Complex	Kappa = -0.874 ~ -0.036	<0.001
Judging whether words are related to cyberbullying victimization	DeepSeek	Simple	Kappa = 0.274	<0.001
	DeepSeek	Complex	Kappa = 0.306	<0.001
	GPT-4o	Simple	Kappa = 0.041	<0.001
	GPT-4o	Complex	Kappa = 0.054	<0.001
Weight assignment	DeepSeek	Simple	ICC = 0.358	<0.001
	DeepSeek	Complex	ICC = 0.280	<0.001
	GPT-4o	Simple	ICC = 0.077	<0.001
	GPT-4o	Complex	ICC = 0.078	<0.001
Dimension classification	DeepSeek	Simple	Kappa = 0.780	<0.001
	DeepSeek	Complex	Kappa = 0.780	<0.001
	GPT-4o	Simple	Kappa = 0.057	<0.001
	GPT-4o	Complex	Kappa = 0.057	<0.001

Judging Whether Initial Words and Synonyms Are Related to Cyberbullying Victimization. DeepSeek showed low consistency with the researchers in judging 719 words related to cyberbullying, while GPT exhibited very low consistency. (see **Table 4**).

Weight Assignment. The intraclass correlation coefficients (ICC) revealed poor consistency between the models and researchers. DeepSeek showed a relatively low consistency, while GPT demonstrated very low consistency (see **Table 4**).

Dimension Classification. DeepSeek demonstrated a relatively high level of agreement with the researchers’ classifications, whereas GPT showed comparatively poorer performance. (see **Table 4**).

#### 3.2.3 Dictionary validation

Cyberbullying Expression Evaluation. In this study, cyberbullying expression was divided into three dimensions based on the lexicon developed in Study 1: harm perception, cyberbullying methods, and coping strategies after being bullied. The consistency results between the models and the researchers are shown in **Table 5**. DeepSeek demonstrated good consistency with the researchers, while GPT’s consistency was mostly at moderate to poor levels. The complex prompt generally outperformed the simple prompt.

**Table 5:** Consistency analysis results between models and researchers (ICC).

Dimension	Model	ICC
Perceived harm	DeepSeek (simple)	0.738***
	DeepSeek (complex)	0.745***
	GPT (simple)	0.426***
	GPT (complex)	0.405***
Cyberbullying methods	DeepSeek (simple)	0.845***
	DeepSeek (complex)	0.850***
	GPT (simple)	0.641***
	GPT (complex)	0.712***
Coping strategies	DeepSeek (simple)	0.770***
	DeepSeek (complex)	0.769***
	GPT (simple)	0.477***
	GPT (complex)	0.535***

*p < 0.05, **p < 0.01, ***p < 0.001.

User Cyberbullying Victimization Level Evaluation. DeepSeek showed good consistency with the researchers (simple: ICC = 0.843, p < 0.001; complex: ICC = 0.846, p < 0.001), while GPT demonstrated moderate consistency (simple: ICC = 0.611, p < 0.001; complex: ICC = 0.634, p < 0.001). The complex prompt performed slightly better than the simple prompt.

Recall Rate and Precision. Using the manual evaluation results as the ground truth, we calculated the average recall rate and precision for the evaluation results of GPT-4o and DeepSeek-R1 in each step to assess their performance. The specific results are shown in **Table 6**. DeepSeek-R1 generally achieved higher recall and precision than GPT-4o, and DeepSeek was able to balance recall and precision, indicating that it can better assist researchers by taking on part of the workload in the lexicon construction process. Although GPT-4o occasionally achieved 100% recall or precision, it was unable to achieve high values for both metrics simultaneously. For example, when evaluating words with a manual assigned weight of 1, GPT (simple) had an average recall rate of 100% and an average precision of 8.23%; GPT (complex) had an average recall rate of 100% and an average precision of 8.26%.

**Table 6:** Average recall rate and precision of evaluation results for each model.

Step		Model	Recall rate (%)		Precision rate (%)	
			M	SD	M	SD
50 Weibo posts		DS (simple)	77.11	7.12	72.50	9.57
		DS (complex)	54.22	11.67	100.00	0.00
		GPT(simple)	21.69	4.67	66.67	0.00
		GPT (complex)	0.00	0.00	0.00	0.00
12,600 Weibo posts		DS(simple)	66.99	19.00	11.79	8.13
		DS (complex)	65.69	17.84	11.53	6.74
		GPT(simple)	29.98	34.90	9.97	15.03
		GPT (complex)	37.19	28.89	26.44	18.27
Weight assignment		DS(simple)	51.43	13.82	46.62	35.49
		DS (complex)	45.49	9.74	41.79	38.55
		GPT(simple)	35.56	48.38	37.13	46.17
		GPT (complex)	35.72	48.26	37.17	46.22
Dimension classification		DS(simple)	76.94	13.57	78.31	12.26
		DS (complex)	77.21	13.94	78.71	11.60
		GPT(simple)	38.17	46.39	74.12	36.98
		GPT (complex)	38.17	46.39	74.12	36.98
Degree of victimization expression	Perceived harm	DS(simple)	45.09	35.12	41.93	35.54
		DS (complex)	48.76	33.74	43.57	34.37
		GPT(simple)	19.70	39.89	12.28	23.19
		GPT (complex)	19.61	39.42	11.63	22.79
	Cyberbullying methods	DS(simple)	46.35	33.21	47.19	32.20
		DS (complex)	44.31	35.32	40.72	38.44
		GPT(simple)	27.66	38.18	34.53	33.88
		GPT (complex)	31.22	42.20	18.42	29.11
	Coping strategies	DS(simple)	43.00	24.54	43.59	35.36
		DS (complex)	45.84	25.07	46.37	34.91
		GPT(simple)	23.30	39.54	16.91	23.69
		GPT (complex)	25.46	39.52	22.60	31.62
Degree of victimization		DS(simple)	46.22	27.46	44.38	35.28
		DS (complex)	47.95	22.33	47.41	36.26
		GPT(simple)	25.67	36.37	33.76	29.01
		GPT (complex)	27.06	37.59	30.28	30.19

### 3.3 Discussion

This study compares the performance of GPT-4o and DeepSeek-R1 in the construction of a Chinese cyberbullying victim lexicon, revealing both the potential and limitations of large language models (LLMs) in assisting lexicon compilation.

In text classification tasks, DeepSeek-R1 generally demonstrated superior performance. Its manually evaluated Kappa coefficient ranges from 0.775 to 0.781 on a 50-microblog subset, whereas GPT-4o achieves 0.575 to 0.650. This disparity likely stems from DeepSeek’s enhanced capability in Chinese language environments (53). For instance, compared to GPT, DeepSeek demonstrates greater stability and applicability in Chinese text adaptation across metrics including lexical richness, word Corrected TTR, word grade standard deviation, modal density, prepositional density, and syntactic complexity (54). Notably, when the dataset size expanded to 12,600 entries, both model types experienced significant performance degradation, suggesting that LLMs may face limitations in semantic generalization when processing large-scale text. However, in certain classification tasks (e.g., the classification of the 1st and 2nd groups within the 12,600 Weibo posts), ChatGPT-4o outperformed DeepSeek-R1. This may be because the classification tasks for these two groups were more sensitive to subtle semantic distinctions and imposed greater demands on contextual understanding, thereby leveraging ChatGPT’s strengths in this regard (81). Prior research likewise suggests that model performance varies across tasks and datasets (81). Therefore, when assisting dictionary construction, the relative advantages and disadvantages of different models should be evaluated according to the specific task requirements and data characteristics rather than assumed to be fixed across contexts.

In the word selection and weight assignment stages, the consistency results of both models were poor. DeepSeek’s Kappa values in the initial word filtering (-0.833 to -0.189) indicate a significant deviation from the manual standard. This may be because lexicon compilation requires deep understanding of sociocultural context and implicit semantic meaning. Compared with humans, LLMs still require improvement in understanding sociocultural context (82). Additionally, previous studies have found that most LLMs perform near or below chance level when detecting implied meanings beyond literal language, suggesting that their ability to interpret more complex, context-dependent pragmatic cues remains limited (83). For example, 84 found that LLMs are easily influenced by the literal meaning of utterances when interpreting discourse meaning. In the comprehension of ironic language, they tend to rely more heavily on the semantic content of the utterance itself and demonstrate insufficient reasoning ability in complex pragmatic scenarios. Furthermore, Zhang et al. (85) systematically evaluated the sarcasm comprehension abilities of current LLMs (including GPT-4 and Claude 3) and found that these models consistently performed worse than supervised pre-trained language model (PLM) baselines across six sarcasm benchmarks. This finding suggests that LLMs still exhibit substantial limitations in tasks requiring semantic understanding beyond literal meaning and more abstract pragmatic reasoning. Interestingly, in the dimension classification task, DeepSeek achieved moderate consistency (Kappa = 0.780). This significant variation in performance across tasks suggests that LLMs may be better suited for highly standardized and structured tasks rather than open-ended tasks requiring creative semantic connections.

The complex prompt performed poorly in certain tasks. For example, in the classification of 50 Weibo posts, the use of complex prompts reduced GPT’s Kappa value from 0.650 to 0.575, and both recall and precision decreased from 21.69 and 66.67 to 0, respectively. Prior studies have similarly found that, in some scenarios, simple prompts outperform complex ones; increasing prompt complexity does not necessarily improve classification performance and may even hinder LLMs from fully focusing on human instructions, thereby degrading performance (86). One possible explanation is that LLMs have limited working memory capacity and exhibit performance ceilings in complex tasks, making it difficult to satisfy multiple constraints simultaneously (87). When prompt complexity exceeds the model’s effective cognitive load in a given task, it can instead lead to performance decline. Meanwhile, some studies have reported improved model performance when role assignment is added to prompts (80), suggesting that the complex prompts used in the present study may still have room for optimization under the current task setting, such as by incorporating few-shot examples or error demonstrations. However, in victimization severity assessment, complex prompts elevated GPT’s ICC from 0.611 to 0.634, indicating that role-assignment strategies may positively influence tasks requiring social cognition. This inconsistency suggests dynamic prompt adaptation mechanisms must be developed for practical applications.

DeepSeek’s balance between recall (maximum 77.11%) and precision (maximum 72.50%) demonstrates its potential as a preprocessing tool for manual screening. Particularly in cyberbullying method identification, where it achieved high consistency (ICC = 0.850), DeepSeek shows practical value in specific dimensions. However, GPT’s extreme performance (e.g., in weight assignment, where recall reached 100% but precision was only 8.26%) warns that strict post-validation mechanisms need to be established in actual use. A hybrid approach is recommended: LLMs for initial screening and dimension classification, and human intervention for weight calibration and review of culturally specific vocabulary.

## 4 Conclusion

This study, for the first time, systematically constructed a Chinese cyberbullying victim dictionary and validated its effectiveness. The results demonstrate that the dictionary has good convergent validity and can effectively identify cyberbullying victim expressions and measure the degree of victimization. Compared to traditional questionnaire surveys, this lexicon enables real-time analysis of social media texts through natural language processing methods, pointing to a potential new pathway for automated detection of cyberbullying victimization. In practical applications, the lexicon may serve as a non-intrusive and low-cost preliminary screening tool for social media platforms, providing technical support for automated warning systems and the targeted delivery of psychological assistance resources. In doing so, it may help shift cyberbullying victim identification from a passive help-seeking model toward proactive detection. In clinical and intervention contexts, with users’ informed consent, the lexicon may also be used to longitudinally monitor changes in social media expressions over time. For example, reductions in the frequency of words within the perceived harm dimension may serve as potential digital behavioral indicators for evaluating the effectiveness of psychological interventions and complementing the subjective bias inherent in traditional self-report measures. However, it should be emphasized that this tool is intended to provide auxiliary information for professional assessment and psychological support, and should not replace clinical judgment or expert human evaluation. Concurrently, the study evaluated the feasibility of GPT-4o and DeepSeek-R1 in assisting dictionary compilation, finding that large language models (LLMs) can effectively reduce manual annotation burdens for specific tasks. They excel at standardized tasks (e.g., dimension division) but exhibit significantly diminished semantic generalization capabilities during large-scale text processing. However, this research still faces several unignorable limitations:

First, the data used in this study were sourced from Sina Weibo, which has unique user demographics and language styles, possibly limiting the generalizability of the findings and the applicability of the cyberbullying victim lexicon to other social platforms, such as QQ, Douyin, RedNote, and Zhihu. Although the present study further conducted an exploratory cross-platform analysis based on textual data from RedNote users and obtained preliminary supportive findings, the representativeness and generalizability of these results remain limited due to the relatively small sample size and restricted platform coverage. In addition, other Chinese social media platforms, such as Zhihu, were not included in the present study. Future research could further optimize and adapt the cyberbullying victim lexicon according to the linguistic characteristics and user expression patterns of different Chinese social media platforms, thereby enhancing its cross-platform generalizability.

Second, because cyberbullying is a negative social experience on social media, some victims may reduce their usage frequency on social platforms after being bullied or even choose to delete content or pause their posts (88), which may affect the accurate recording of their psychological state. This could lead to data bias, with research findings reflecting the psychological characteristics of individuals who continue to express themselves. Future research could use cross-platform tracking or a mixed-method approach (online and offline) to supplement data from individuals who have reduced expression, providing a more representative sample.

Third, there may be sample bias, as the victim group in Study 1 had a higher proportion of women (68.6%). This may be related to the greater likelihood of females experiencing cyberbullying compared with males (19, 89). However, males and females differ in their coping strategies, perceived harm, and types of cyberbullying experienced (90–92). For example, regarding coping strategies, females tend to adopt ‘support seeking’, whereas males are more likely to engage in ‘direct reaction’ (22). A female-dominated sample may therefore lead the lexicon to include more support-seeking-related terms in this dimension while underrepresenting direct-reaction expressions (e.g., retaliation or constructive feedback). This may reduce the external validity of the lexicon, and caution should be exercised when applying it to male populations. Future studies may consider recruiting cyberbullying victims of different genders to examine whether the lexicon’s applicability differs significantly across gender groups.

Fourth, in Study 1, the researchers’ judgments regarding whether users met the inclusion criteria for the victim group and the assignment of word weights were based on the detailed training they received on the definition, types, and manifestations of cyberbullying. Although a majority-voting approach was adopted, explicit objective evaluation criteria were still lacking. As a result, the researchers’ judgments were inevitably influenced by subjective cognition, which may have reduced the objectivity of the assessment to some extent.

Fifth, during the lexicon construction and validation process, only 3–4 expert raters were recruited for manual evaluation and judgment. Although this approach is consistent with previous studies on text annotation and lexicon construction (36, 93), and the expert raters in the present study demonstrated good inter-rater reliability, the relatively limited number of experts may still restrict the stability and external generalizability of the evaluation results to some extent. Future studies may consider recruiting larger and more diverse expert panels to further improve the robustness of the evaluation results and the generalizability of the lexicon.

Sixth, although the textual dataset used in this study was relatively large, the number of confirmed cyberbullying victims remained limited (N = 35). This was partly because identifying genuine victims in social media environments requires relatively stringent screening criteria to ensure labeling validity. Such a strategy helps enhance the internal validity of the study but may also limit the generalizability of the findings to some extent. Nevertheless, from a text-mining perspective, the representativeness of linguistic features primarily depends on the scale of the corpus and its coverage of linguistic variations within the target population (94), rather than solely on the number of users. The present study included more than 10,000 Weibo posts from cyberbullying victims, which enabled broad coverage of linguistic expression patterns related to cyberbullying victimization. In addition, previous lexicon-based studies have demonstrated that even with relatively limited target populations, robust and interpretable linguistic features can still be extracted when sufficient textual data are available (36, 95). Future research could further enhance the generalizability of the findings by expanding the sample size, integrating multi-platform data, and conducting external validation in more diverse populations.

Seventh, the performance evaluation of LLM models in Study 2 relied on human assessment results as the reference standard, overlooking potential limitations inherent in human evaluation. Furthermore, the models exhibited extremely low consistency levels in certain large-scale text processing tasks, possibly due to ambiguities in task design or model instructions. This study failed to conduct supplementary qualitative analysis to identify underlying causes, which may diminish the guidance value of its conclusions for subsequent LLM optimization or evaluation scheme development.

Finally, the complex prompt in this study only involved role assignment. Future studies could optimize prompts by incorporating small-sample training, error examples, and chain-of-thought reasoning to further improve model performance.

## Data Availability

The original contributions presented in the study are included in the article/**Supplementary Material**. Further inquiries can be directed to the corresponding authors.
