# Supplementary material for: Cyberbullying victimization identification and large language model-assisted assessment: a study of cyberbullying victimization lexicon construction and validation
Source: Front Psychiatry. 2026 Jun 24;17:1826042. doi: 10.3389/fpsyt.2026.1826042 (PMC13341625; doi:10.3389/fpsyt.2026.1826042)
Supplement: Supplementary file 1 [file Supplementaryfile1.docx]

**Appendix 1**

Below are the simple and complex prompts for GPT and DeepSeek. Items 1-4 provide definitions and explanations, used in every step; A–H represent task instructions that were separately input to GPT and DeepSeek across different subtasks. Each subtask used only its corresponding single instruction (A/B/C/…/H), rather than all instructions simultaneously (see Supplementary Table 1). For example, in the DeepSeek (simple prompt) task of evaluating the subset of 50 Weibo posts, the instruction content consisted of Items ‘1–4’ and ‘A’ from the simple prompt.

Supplementary Table 1. Task Instructions (A-H) Used in Each Subtask

| Subtask Content | Task Instruction |
| --- | --- |
| Determine whether 50 Weibo posts constitute cyberbullying victimization texts | A |
| Determine whether approximately 3,100 Weibo posts constitute cyberbullying victimization texts | B |
| Extract relevant words from cyberbullying victimization–related Weibo posts, scales, and dictionaries | C |
| Determine whether words are related to cyberbullying victimization | D |
| Assign word weights | E |
| Classify word dimensions | F |
| Evaluate 500 Weibo posts across three dimensions | G |
| Assess the degree of cyberbullying victimization reflected in 500 Weibo posts | H |

**Simple prompt：**

1. Definition of Cyberbullying: Cyberbullying is defined as the use of digital technologies to target one or more individuals, where the victim perceives it as a deliberate and harmful act. When the victim not only perceives the intention behind the bullying but also feels harmed by it, they become a cyberbullying victim.

2. Types of Cyberbullying: These include flaming, harassment, stalking, exclusion, impersonation, catfishing, trolling, hacking, sexting, and outing & trickery. Detailed descriptions of these types are as follows:

①Flaming refers to the use of harmful language in emails, text messages, or online chatrooms, often involving online battles or short, intense exchanges between two or more individuals.

②Harassment involves the regular dissemination of insulting, hateful, or threatening messages.

③Stalking refers to online tracking of individuals, sending them emails or text messages with the intention to intimidate or harm.

④Exclusion is the deliberate act of excluding someone from a group or community.

⑤Impersonation involves pretending to be someone else online and publishing correct or misleading information about them to damage their reputation.

⑥Catfishing refers to the act of creating a fake online identity to deceive, abuse, or trick specific victims.

⑦Trolling is the intentional posting of provocative or insulting comments to upset others, with the aim of provoking, disturbing, or manipulating others' emotions.

⑧Hacking is the unauthorized access to a person's social media account to publish inappropriate content and damage their reputation.

⑨Sexting involves the exchange of explicit photos or messages via digital devices, often to intimidate or humiliate the recipient.

⑩Outing & Trickery involves stealing personal information without consent and sharing it electronically to harm or embarrass the individual.

3. Victim Impact: Cyberbullying can lead to reduced happiness, heightened negative emotions, and adverse changes in moral perceptions and personality traits. Specific expressions of these impacts may include:

①Negative emotional expression: "I feel worthless." "I'm so tired every day and don’t want to do anything." "Why are they doing this to me? I can’t take it anymore."

②Self-doubt and low self-esteem: "Am I really this bad?" "It feels like nothing I do is right." "Why am I always targeted?"

③Expression of loneliness and helplessness: "No one truly understands me." "It feels like the whole world is against me." "Being alone is so hard."

④Pessimism about the future: "I don’t know what’s going to happen, everything feels dark." "I can’t see any hope." "I feel like I’ll never be happy again."

⑤Seeking support and understanding: "I really hope someone will talk to me." "I need someone I can rely on." "Can someone tell me what to do?"

⑥Increased perception of moral unfairness: "I just told the truth, why am I being treated like this? This injustice breaks my heart." "Every time I see those who attack me go unpunished, I feel the world is so unfair."

⑦Increased perception of moral impurity: "The filthy language online really disgusts me. How did the world become so vile?" "The rumors and malicious attacks spread by people make me feel the world is so corrupt."

⑧Decreased agreeableness: "After so many attacks and betrayals, I’ve lost all faith in humanity." "I can’t trust anyone anymore, they’re all two-faced."

⑨Decreased extraversion: "I just want to be alone, I don’t want to interact with anyone." "I used to enjoy parties, but now socializing feels exhausting, I prefer staying home." "I’ve decided to delete all my social media accounts, no more online interaction."

⑩Decreased conscientiousness: "After all this online abuse, I can’t even get motivated for work, everything seems meaningless." "I’ve been attacked so much, I can’t focus on completing any tasks."

⑪Increased neuroticism: "Every time someone comments on my post, I feel anxious, fearing another attack." "I can’t sleep, all I think about are those cruel comments, I feel like I’m going crazy."

Note: The above statements are examples of expressions that victims of cyberbullying may exhibit after experiencing such abuse.

4. Criteria for Selecting Cyberbullying Victim Posts and Vocabulary: Based on the definitions, types, and impacts on victims outlined above, the expressions of cyberbullying victims are likely to involve three aspects: (1) specific cyberbullying behaviors or methods that the victim perceives as deliberate actions by the perpetrator; (2) the victim's perception of harm; and (3) coping strategies after experiencing cyberbullying.

A. There are 50 Weibo posts here. Based on the definitions, types, potential impacts on victims, and criteria for selecting cyberbullying victim posts and vocabulary outlined above, combined with the context of cyberbullying victims' expressions on social media, please assess whether each post is likely to be an expression of a cyberbullying victim. If you think the post represents the expression of a cyberbullying victim, label it as 1; otherwise, label it as 0. Please present the results in a table.

B. There are approximately 3,100 Weibo posts here. Based on the definitions, types, potential impacts on victims, and criteria for selecting cyberbullying victim posts and vocabulary outlined above, combined with the context of cyberbullying victims' expressions on social media, please assess whether each post is likely to be an expression of a cyberbullying victim. If you think the post represents the expression of a cyberbullying victim, label it as 1; otherwise, label it as 0. Please present the results in a table.

C. There are 132 Weibo posts, 5 cyberbullying-related scales, and 3 cyberbullying victim-related dictionaries here. Based on the definitions, types, potential impacts on victims, and criteria for selecting cyberbullying victim posts and vocabulary outlined above, combined with the context of cyberbullying victims' expressions on social media, please select words from each Weibo post, scale, and dictionary that are likely to be used by cyberbullying victims when expressing their experience of cyberbullying on social media. Please present the results in a table.

D. There are 719 words here. Based on the definitions, types, potential impacts on victims, and criteria for selecting cyberbullying victim posts and vocabulary outlined above, combined with the context of cyberbullying victims' expressions on social media, please assess whether each word is likely to be used by cyberbullying victims when expressing their experience of cyberbullying on social media. If you think the word is relevant, label it as 1; otherwise, label it as 0. Please present the results in a table.

E. There are 521 words here. Based on the definitions, types, potential impacts on victims, and criteria for selecting cyberbullying victim posts and vocabulary outlined above, combined with the context of cyberbullying victims' expressions on social media, please score each word on a scale from 1 to 3, where a higher score indicates a greater relevance to cyberbullying victimization. Please present the results in a table that includes the 521 words and their corresponding scores (1-3).

F. There are 521 words here. Based on the definitions, types, potential impacts on victims, and criteria for selecting cyberbullying victim posts and vocabulary outlined above, combined with the context of cyberbullying victims' expressions on social media, please determine which of the following three dimensions each word belongs to: "Cyberbullying Methods," "Harm Perception," or "Coping Strategies." When an individual experiences cyberbullying, they may first describe the cyberbullying behaviors or methods they suffered, followed by the harms caused by those behaviors or methods, and possibly also describe the coping strategies they employed. Please classify each word according to these dimensions. If you think the word relates to describing cyberbullying methods, label it as 1; if it relates to harm perception, label it as 2; if it relates to coping strategies, label it as 3. Please present the results in a table.

G. There are 500 Weibo posts here. Based on the definitions, types, potential impacts on victims, and criteria for selecting cyberbullying victim posts and vocabulary outlined above, combined with the context of cyberbullying victims' expressions on social media, please evaluate the "Cyberbullying Methods," "Harm Perception," and "Coping Strategies" presented in each post. Use a Likert 5-point scale, where 1 means "this post is unrelated to the dimension," and 5 means "this post is highly related to the dimension." A higher score indicates that the post expresses more of the corresponding victim's experience in that dimension. Please present the results in the same table.

H. There are 500 Weibo posts here. Based on the definitions, types, potential impacts on victims, and criteria for selecting cyberbullying victim posts and vocabulary outlined above, combined with the context of cyberbullying victims' expressions on social media, please assess the level of cyberbullying victimization expressed by each user. Use a Likert 5-point scale, where 1 means "the user's expression of cyberbullying victimization is very low," and 5 means "the user's expression of cyberbullying victimization is very high." A higher score indicates that the post expresses a higher level of cyberbullying victimization. Please present the results in the same table.

**Complex prompt:**

1. Definition of Cyberbullying: Cyberbullying is defined as the use of digital technologies to target one or more individuals, where the victim perceives it as a deliberate and harmful act. When the victim not only perceives the intention behind the bullying but also feels harmed by it, they become a cyberbullying victim.

2. Types of Cyberbullying: These include flaming, harassment, stalking, exclusion, impersonation, catfishing, trolling, hacking, sexting, and outing & trickery. Detailed descriptions of these types are as follows:

①Flaming refers to the use of harmful language in emails, text messages, or online chatrooms, often involving online battles or short, intense exchanges between two or more individuals.

②Harassment involves the regular dissemination of insulting, hateful, or threatening messages.

③Stalking refers to online tracking of individuals, sending them emails or text messages with the intention to intimidate or harm.

④Exclusion is the deliberate act of excluding someone from a group or community.

⑤Impersonation involves pretending to be someone else online and publishing correct or misleading information about them to damage their reputation.

⑥Catfishing refers to the act of creating a fake online identity to deceive, abuse, or trick specific victims.

⑦Trolling is the intentional posting of provocative or insulting comments to upset others, with the aim of provoking, disturbing, or manipulating others' emotions.

⑧Hacking is the unauthorized access to a person's social media account to publish inappropriate content and damage their reputation.

⑨Sexting involves the exchange of explicit photos or messages via digital devices, often to intimidate or humiliate the recipient.

⑩Outing & Trickery involves stealing personal information without consent and sharing it electronically to harm or embarrass the individual.

3. Victim Impact: Cyberbullying can lead to reduced happiness, heightened negative emotions, and adverse changes in moral perceptions and personality traits. Specific expressions of these impacts may include:

①Negative emotional expression: "I feel worthless." "I'm so tired every day and don’t want to do anything." "Why are they doing this to me? I can’t take it anymore."

②Self-doubt and low self-esteem: "Am I really this bad?" "It feels like nothing I do is right." "Why am I always targeted?"

③Expression of loneliness and helplessness: "No one truly understands me." "It feels like the whole world is against me." "Being alone is so hard."

④Pessimism about the future: "I don’t know what’s going to happen, everything feels dark." "I can’t see any hope." "I feel like I’ll never be happy again."

⑤Seeking support and understanding: "I really hope someone will talk to me." "I need someone I can rely on." "Can someone tell me what to do?"

⑥Increased perception of moral unfairness: "I just told the truth, why am I being treated like this? This injustice breaks my heart. "Every time I see those who attack me go unpunished, I feel the world is so unfair."

⑦Increased perception of moral impurity: "The filthy language online really disgusts me. How did the world become so vile?" "The rumors and malicious attacks spread by people make me feel the world is so corrupt."

⑧Decreased agreeableness: "After so many attacks and betrayals, I’ve lost all faith in humanity." "I can’t trust anyone anymore, they’re all two-faced."

⑨Decreased extraversion: "I just want to be alone, I don’t want to interact with anyone." "I used to enjoy parties, but now socializing feels exhausting, I prefer staying home." "I’ve decided to delete all my social media accounts, no more online interaction."

⑩Decreased conscientiousness: "After all this online abuse, I can’t even get motivated for work, everything seems meaningless." "I’ve been attacked so much, I can’t focus on completing any tasks."

⑪Increased neuroticism: "Every time someone comments on my post, I feel anxious, fearing another attack." "I can’t sleep, all I think about are those cruel comments, I feel like I’m going crazy."

Note: The above statements are examples of expressions that victims of cyberbullying may exhibit after experiencing such abuse.

4. Criteria for Selecting Cyberbullying Victim Posts and Vocabulary: Based on the definitions, types, and impacts on victims outlined above, the expressions of cyberbullying victims are likely to involve three aspects: (1) specific cyberbullying behaviors or methods that the victim perceives as deliberate actions by the perpetrator; (2) the victim's perception of harm; and (3) coping strategies after experiencing cyberbullying.

A. You are a researcher working on creating a cyberbullying victimization dictionary. There are 50 Weibo posts here. Based on the definitions, types, potential impacts on victims, and criteria for selecting cyberbullying victim posts and vocabulary outlined above, combined with the context of cyberbullying victims' expressions on social media, please assess whether each post is likely to be an expression of a cyberbullying victim. If you think the post represents the expression of a cyberbullying victim, label it as 1; otherwise, label it as 0. Please present the results in a table.

B. You are a researcher working on creating a cyberbullying victimization dictionary. There are approximately 3,100 Weibo posts here. Based on the definitions, types, potential impacts on victims, and criteria for selecting cyberbullying victim posts and vocabulary outlined above, combined with the context of cyberbullying victims' expressions on social media, please assess whether each post is likely to be an expression of a cyberbullying victim. If you think the post represents the expression of a cyberbullying victim, label it as 1; otherwise, label it as 0. Please present the results in a table.

C. You are a researcher working on creating a cyberbullying victimization dictionary. There are 132 Weibo posts, 5 cyberbullying-related scales, and 3 cyberbullying victim-related dictionaries here. Based on the definitions, types, potential impacts on victims, and criteria for selecting cyberbullying victim posts and vocabulary outlined above, combined with the context of cyberbullying victims' expressions on social media, please select words from each Weibo post, scale, and dictionary that are likely to be used by cyberbullying victims when expressing their experience of cyberbullying on social media. Please present the results in a table.

D. You are a researcher working on creating a cyberbullying victimization dictionary. There are 719 words here. Based on the definitions, types, potential impacts on victims, and criteria for selecting cyberbullying victim posts and vocabulary outlined above, combined with the context of cyberbullying victims' expressions on social media, please assess whether each word is likely to be used by cyberbullying victims when expressing their experience of cyberbullying on social media. If you think the word is relevant, label it as 1; otherwise, label it as 0. Please present the results in a table.

E. You are a researcher working on creating a cyberbullying victimization dictionary. There are 521 words here. Based on the definitions, types, potential impacts on victims, and criteria for selecting cyberbullying victim posts and vocabulary outlined above, combined with the context of cyberbullying victims' expressions on social media, please score each word on a scale from 1 to 3, where a higher score indicates a greater relevance to cyberbullying victimization. Please present the results in a table that includes the 521 words and their corresponding scores (1-3).

F. You are a researcher working on creating a cyberbullying victimization dictionary. There are 521 words here. Based on the definitions, types, potential impacts on victims, and criteria for selecting cyberbullying victim posts and vocabulary outlined above, combined with the context of cyberbullying victims' expressions on social media, please determine which of the following three dimensions each word belongs to: "Cyberbullying Methods," "Harm Perception," or "Coping Strategies." When an individual experiences cyberbullying, they may first describe the cyberbullying behaviors or methods they suffered, followed by the harms caused by those behaviors or methods, and possibly also describe the coping strategies they employed. Please classify each word according to these dimensions. If you think the word relates to describing cyberbullying methods, label it as 1; if it relates to harm perception, label it as 2; if it relates to coping strategies, label it as 3. Please present the results in a table.

G. You are a researcher working on creating a cyberbullying victimization dictionary. There are 500 Weibo posts here. Based on the definitions, types, potential impacts on victims, and criteria for selecting cyberbullying victim posts and vocabulary outlined above, combined with the context of cyberbullying victims' expressions on social media, please evaluate the "Cyberbullying Methods," "Harm Perception," and "Coping Strategies" presented in each post. Use a Likert 5-point scale, where 1 means "this post is unrelated to the dimension," and 5 means "this post is highly related to the dimension." A higher score indicates that the post expresses more of the corresponding victim's experience in that dimension. Please present the results in the same table.

H. You are a researcher working on creating a cyberbullying victimization dictionary. There are 500 Weibo posts here. Based on the definitions, types, potential impacts on victims, and criteria for selecting cyberbullying victim posts and vocabulary outlined above, combined with the context of cyberbullying victims' expressions on social media, please assess the level of cyberbullying victimization expressed by each user. Use a Likert 5-point scale, where 1 means "the user's expression of cyberbullying victimization is very low," and 5 means "the user's expression of cyberbullying victimization is very high." A higher score indicates that the post expresses a higher level of cyberbullying victimization. Please present the results in the same table.
